# The prevalence of asthma and allergic rhinitis in Nigeria: A nationwide survey among children, adolescents and adults

**DOI:** 10.1371/journal.pone.0222281

**Published:** 2019-09-13

**Authors:** Obianuju B. Ozoh, Sunday A. Aderibigbe, Adaeze C. Ayuk, Olufemi O. Desalu, Olufela E. Oridota, Olajumoke Olufemi, Eruke Egbagbe, Musa Babashani, Azeezat Shopeyin, Kingsley Ukwaja, Sandra K. Dede

**Affiliations:** 1 Department of Medicine, College of Medicine, University of Lagos, Lagos State, Nigeria; 2 Lagos University Teaching Hospital, Lagos State, Nigeria; 3 Department of Public Health, College of Health Sciences, University of Ilorin, Kwara State, Nigeria; 4 Department of Paediatrics, College of Medicine, University of Nigeria Teaching Hospital, Enugu State, Nigeria; 5 Department of Medicine, College of Health Sciences, University of Ilorin, Kwara State, Nigeria; 6 Department of Community Medicine and Child Health, College of Medicine, University of Lagos, Lagos State, Nigeria; 7 Department of Medicine, College of Medicine, University of Benin, Edo State, Nigeria; 8 Department of Medicine, College of Medicine, Aminu Kano University, Kano State, Nigeria; 9 Department of Internal Medicine, Federal Teaching Hospital Abakiliki, Ebonyi State, Nigeria; UCIBIO-REQUIMTE, Faculty of Pharmacy, University of Porto, PORTUGAL

## Abstract

**Purpose:**

Asthma is an important cause of morbidity and mortality worldwide and information on the prevalence of asthma in Nigeria is inconsistent. Nationally representative data, important for health planning is unavailable. We aimed to determine the current prevalence of asthma and allergic rhinitis in Nigeria.

**Materials and methods:**

A cross-sectional population survey conducted between June 2017 and March 2018 across five cities representing five geo-political zones in Nigeria. Validated screening questionnaires were used to identify persons with asthma and allergic rhinitis respectively. Asthma was defined as physician diagnosed asthma, clinical asthma and by presence of wheeze in the last 12 months respectively. Socio-demographic information, tobacco smoking, sources of household cooking fuel were also obtained.

**Results:**

A total of 20063 participants from 6024 households were screened. The prevalence (95% confidence interval) of physician diagnosed asthma, clinical asthma and wheeze was 2.5% (2.3–2.7%), 6.4% (6.0–6.64%) and 9.0% (8.6–9.4%) respectively. The prevalence of allergic rhinitis was 22.8% (22.2–23.4%). The prevalence of asthma and rhinitis increased with age (prevalence of clinical asthma: 3.1% (2.8–3.4%), 9.8% (9.1–10.5) and 10.7% (9.4%-12.0) among 6–17 years, 18–45 years and >45 years respectively). Prevalence also varied across different cities with the highest prevalence of clinical asthma occurring in Lagos (8.0%) and the lowest in Ilorin (1.1%). The frequency of allergic rhinitis among persons with clinical asthma was 74.7%. Presence of allergic rhinitis, family history of asthma, current smoking and being overweight were independent determinants of current asthma among adults.

**Conclusion:**

The prevalence of asthma and allergic rhinitis in Nigeria is high with variabilities across regions and age groups. The number of persons with clinical asthma in Nigeria (approximately 13 million) is likely to rank among the highest in Africa. This warrants prioritization by stakeholders and policy makers to actively implement risk reduction measures and increase investment in capacity building for the diagnosis and treatment of asthma and allergic rhinitis.

## Introduction

Asthma is an important cause of morbidity and mortality worldwide, ranking high as a cause of disability adjusted life years (DALYs) in 2015 [1]. The Global Burden of Diseases (GBD) project estimates that the prevalence of asthma increased by about 12% globally between 2005 and 2015, mostly in developing countries [2,3]. Economic development and urbanization in many parts of Africa for example are likely to contribute to the upsurge in the prevalence of asthma in this region. Urbanization has led to increased income, adoption of the Western diet and lifestyle, decline in childhood infections, atopic sensitization and increase in air pollution which are associated with developing asthma [4–7].

Derivation of overall asthma burden combines information from direct enumeration of a representative population with data related to morbidity and mortality. Therefore, further availability of broad-based and representative prevalence data is desirable to more accurately estimate the burden of asthma, to guide future projections, health service planning, allocation of resources and to inform policy.

Allergic rhinitis is an important cause of morbidity and shares common pathophysiology with asthma, often co-existing with asthma [8]. The presence of allergic rhinitis is a risk factor for adult onset asthma and its co-existence with asthma is usually associated with poor asthma control [9]. The prevalence of allergic rhinitis in a population also reflects the level of atopic sensitization in that population and directs intervention.

Few studies have evaluated the population-based prevalence of asthma in sub-Saharan Africa [10]. Nigeria is one of the fastest growing populations in the world and the largest in Africa (with a population estimate of about 198 million) [11]. At present, there is an absence of nationally representative asthma prevalence estimate derived from a community survey in Nigeria. Previous studies have been limited by sample population, geographical distribution and non-uniformity in asthma definition precluding the estimation of the national asthma prevalence [12–17]. Nigeria is a diverse country with heterogeneity in climate, ethnicity, urbanization and cultural practices making it expedient to derive a national estimate from a broad-based survey. These factors also influence the prevalence of allergic rhinitis and very few studies have explored the co-existence of asthma and allergic rhinitis.

The Asthma Insight and Reality (AIR) survey is a worldwide research initiative that used a standardized protocol to screen a nationally representative sample of the population to identify persons with asthma [18–20]. The screening questionnaire for asthma in the AIR survey was validated and similar to those of the American Thoracic Society (ATS), the European Community Respiratory Health Survey (ECRHS) questionnaire and the International Study of Asthma and Allergies in Childhood (ISAAC) [3,21–22]. The AIR surveys were conducted in Europe, United States of America and other parts of the world but not in sub-Saharan Africa [18–20].

This present study is the first AIR survey in sub-Saharan Africa and in the absence of a nationally representative prevalence data for asthma in Nigeria, it is imperative to first quantify the national prevalence of asthma. The aim of this study is to determine the national prevalence of asthma and allergic rhinitis respectively in Nigeria among children, adolescents and adults using validated screening questionnaires and to determine variations across age groups and regions. This study is different from previous studies in that we surveyed a very large nationally and geographically representative sample population, that included most age groups, and we used a consistent method in the definition of asthma and allergic rhinitis that enabled the estimation of a national prevalence and also comparisons across cities and age groups. Identification of these variations enhance understanding of the burden of asthma and rhinitis and may suggest potential environmental risks. It also guides prioritization, health system planning and allocation of scarce health resources across regions and age groups. We obtained the prevalence of asthma using three operational definitions: physician diagnosed asthma, current asthma (which includes treated asthma) and the presence of wheeze in the preceding 12 months. Diagnosis of allergic rhinitis was based on the validated Score For Allergic Rhinitis which utilizes a robust combination of symptoms, past medical history and family history. We also compared the characteristics of persons with asthma and persons without asthma and determined independent risk factors for asthma.

## Methods

This study was a cross-sectional population-based survey conducted across five cities in Nigeria between June 2017 and March 2018.

### Ethical considerations

We obtained approval of the study protocol from the Health Research Ethics Committees of all participating institutions and from the National Health Research Ethics Committee of the Federal Ministry of Health, Nigeria. Additional permissions were obtained from the state Ministries of Health and from the selected Local Government Area (LGA) health authorities. Community entry protocol at all sites involved notification of the Local Community Development Associations (LCDA), obtaining permissions from community leaders and key stakeholders for the creation of awareness prior to study commencement. We disseminated notifications of the intended study dates to the communities through the LGA representatives prior to study commencement for community sensitization. We obtained informed consent from the head of each household for initial access into the home and also from individual participants for adults. For children less than 18 years of age, we obtained assent and permission from the parents or guardian prior to interview.

### Study sites and study participants

Sampling and interviewing limitations already identified in the previous AIR surveys required that the sampling frame be restricted to urban areas within countries as per protocol [18]. Nigeria is divided into six zones referred to as geo-political zones based on geographic location, culture, ethnicity and common history. We selected five urban cities, one from each geopolitical zone based on the cosmopolitan nature and the current United Nations population estimates [11]. Two cosmopolitan cities with the largest populations in Nigeria: Lagos (South West) and Kano (North West) were selected. We also selected Ilorin (North Central), Enugu (South East) and Benin-city (South South). We did not include any city from the North-East zone of the country due to security challenges and internal displacement of part of the population in that region at the time of the study.

Study participants were children and adolescents 6 years to 17 years of age and adults ≥18 years who resided in selected households. The institutional population that were living in prisons, hospitals and school dormitories at that time were excluded.

### Recruitment and Questionnaire administration

**Fig 1:**
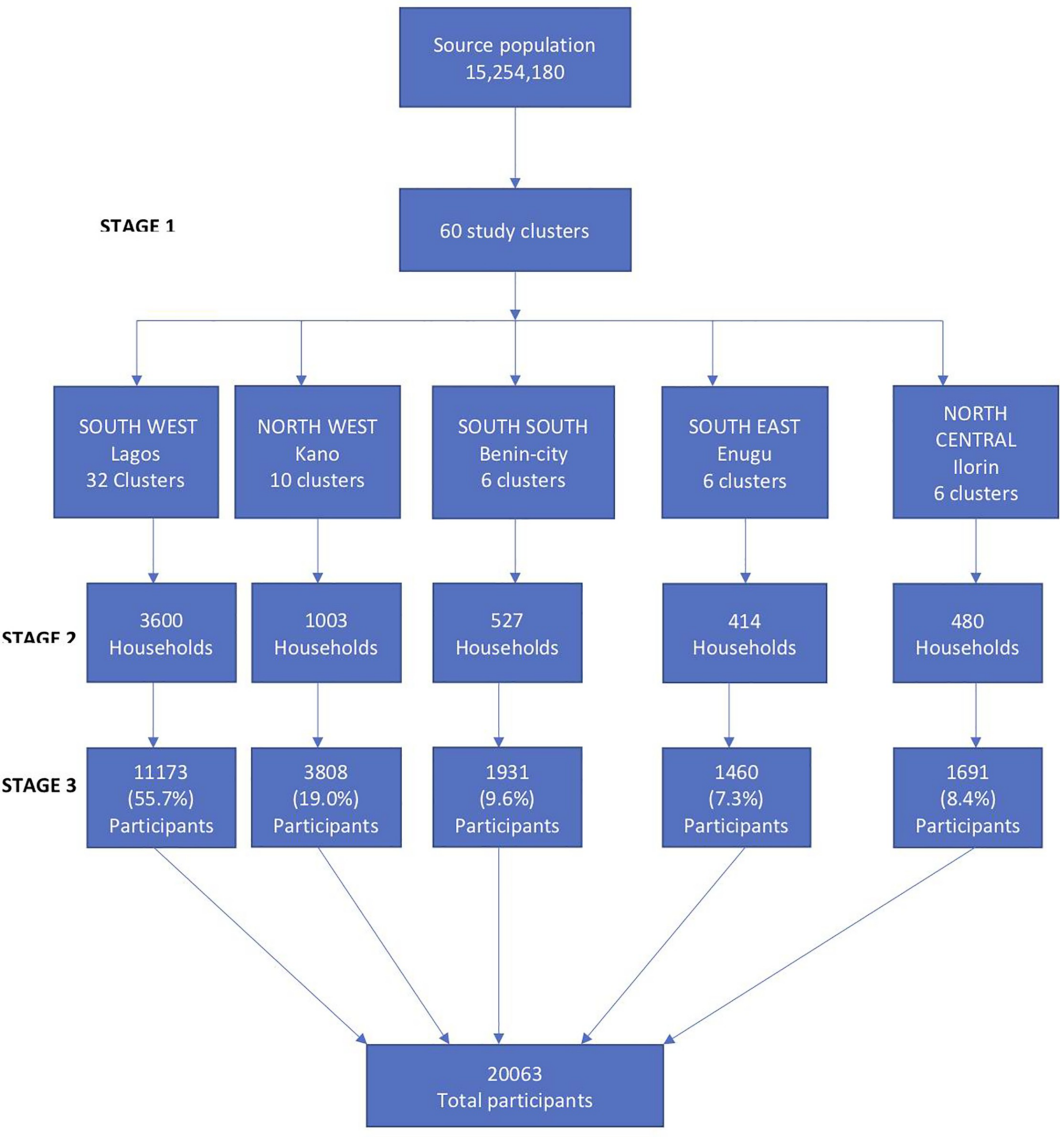
Multistage sampling approach for participant selection. Source population is the number of participants across the five selected cities based on the current population estimate.

The AIR survey sampling plan was designed to provide a nationally representative sample of households for screening to identify a community prevalence of persons with asthma and also to identify persons with asthma for further evaluation on asthma management. We used a stratified multistage sampling approach to select households using the number of Enumeration Areas (EAs) in the urban areas in each city (Fig 1).

The EAs are operational segments defined for the 2006 census enumeration, each comprising about 37 households in urban areas [23]. The EAs in the designated urban areas in each city were the primary sampling unit from which the EAs for the study were randomly selected. The households in each EA were the secondary sampling units from which all eligible household members present at the time of visit were screened for asthma. Screening was conducted in the evenings on weekdays from 4 pm to 7 pm and on weekends and public holidays from 9 am to 7 pm.

We estimated that screening household members in 5000 households will identify a community prevalence of persons with asthma as well as a representative sample of persons with asthma for further evaluation. This was based on a 10% prevalence of asthma as reported in two previous meta-analysis for the prevalence of asthma in Nigeria and Africa respectively, an effect size of 2 for cluster sampling at 95% confidence level [10,24]. Households were distributed across the five cities by proportion using the estimated population of persons residing in each city at that time [11]. Approximately, 59% of the total eligible participants resided in the city of Lagos, 24% in Kano, 7.4% in Benin, 5.3% in Ilorin and 4.5% in Enugu.

We screened all eligible participants in selected households for asthma and allergic rhinitis using structured questionnaires by trained interviewers. The questionnaires were translated and back translated from English into the three major local Nigerian languages (Hausa, Igbo, Yoruba) using a standard protocol and interviews were conducted within the households in English or any of the local languages. The questionnaires were completed electronically on an electronic data collection system the Open Data Kit (ODK)® installed on mobile phones and uploaded to a secure server at the end of each day after review by the study supervisor. The use of the electronic data collection was to the improve accuracy of data gathering and data entry and minimize missing data. We pre-tested the questionnaire in a non-participating EA in Lagos prior to the start of the study and made all necessary adjustments on the wording and skip logic pattern on the ODK® for more accurate data capture.

The questions for asthma screening were similar to previous AIR studies [18]. The five-item questionnaire obtained information on previous physician diagnosis of asthma and presence of any of the following in the preceding 12 months: asthma symptoms (wheeze, chest tightness, night symptoms), asthma attack, use of asthma medication(s)and use of any of the listed commonly available asthma medications for symptom relief. Presence of allergic rhinitis was assessed using the Score for Allergic Rhinitis (SFAR) questionnaire; a validated eight-item questionnaire that obtains information on nasal symptoms, periodicity (perennial versus seasonal), associated conjunctivitis, specific triggers, allergic status, results of allergy test, allergy diagnosis and family history of atopy [25]. A score of ≥7 (maximum score 16) on the SFAR questionnaire was considered positive for allergic rhinitis. We also obtained socio-demographic information: age, gender, tobacco smoking, level of education, household income and source of household cooking fuel and measured height and weight by standard methods to calculate the body mass index in kilogram/m^2^[26]. The body mass index was categorized as follows: Underweight (<18 kg/m^2^), normal (18–25 kg/m^2^), overweight (26–29 kg/m^2^), obese (≥30 kg/m^2^) [26].

### Operational definitions for asthma and statistical analysis

Operational definitions for asthma were similar to those used in the World Health Survey (WHS) for asthma prevalence [27], but we considered the use of asthma medication within 12 months. Asthma was defined as follows:

*Physician diagnosed asthma*: previous physician diagnosis of asthma.

*Clinical asthma*: physician diagnosed asthma and/or affirmation to the presence of asthma symptoms or asthma attack and the use of asthma medications.

*Wheeze in the last 12 months*: symptoms within the preceding 12 months of ‘wheeze’ or ‘whistling noise while breathing’ related to a notable trigger such as aeroallergen and usually worse at night.

We calculated population size weights, age and gender distribution weights for each participant represented in the study. The sampling weights were then used to calculate population-based prevalence estimates at 95% confidence interval across categories of the independent variables and compared the prevalence of asthma across cities. The participants were categorized into children (6–17 years), adults (18–45 years) and older adults (>45 years). To enable comparison with the ISAAC study, we also sub-categorized children and adolescents into those that are 6–7 years and 13–14 years and determined the prevalence of asthma among these age groups. In addition, the prevalence of asthma among adolescents (10–19 years) as defined by the World Health Organization (WHO) was also determined to highlight the prevalence in this vulnerable age group in whom asthma is among the top ten causes of chronic illness and disability [28]. We assessed for the independent determinants of clinical asthma among adults using a multivariate logistic regression model. Data analysis was performed using STATA/SE 13.0.

## Results

The average household response rate across all cities was 97.8%, (98.6%, 98.4%, 98.5%, 97.2% and 96.1% for Lagos, Kano, Benin city, Ilorin and Enugu respectively). A total of 6,024 households (3600, 1003, 527, 480 and 414 from Lagos, Kano, Benin city, Ilorin and Enugu respectively) and 20063 participants were screened from 60 EA clusters across all five cities (Fig 1).

**Fig 2:**
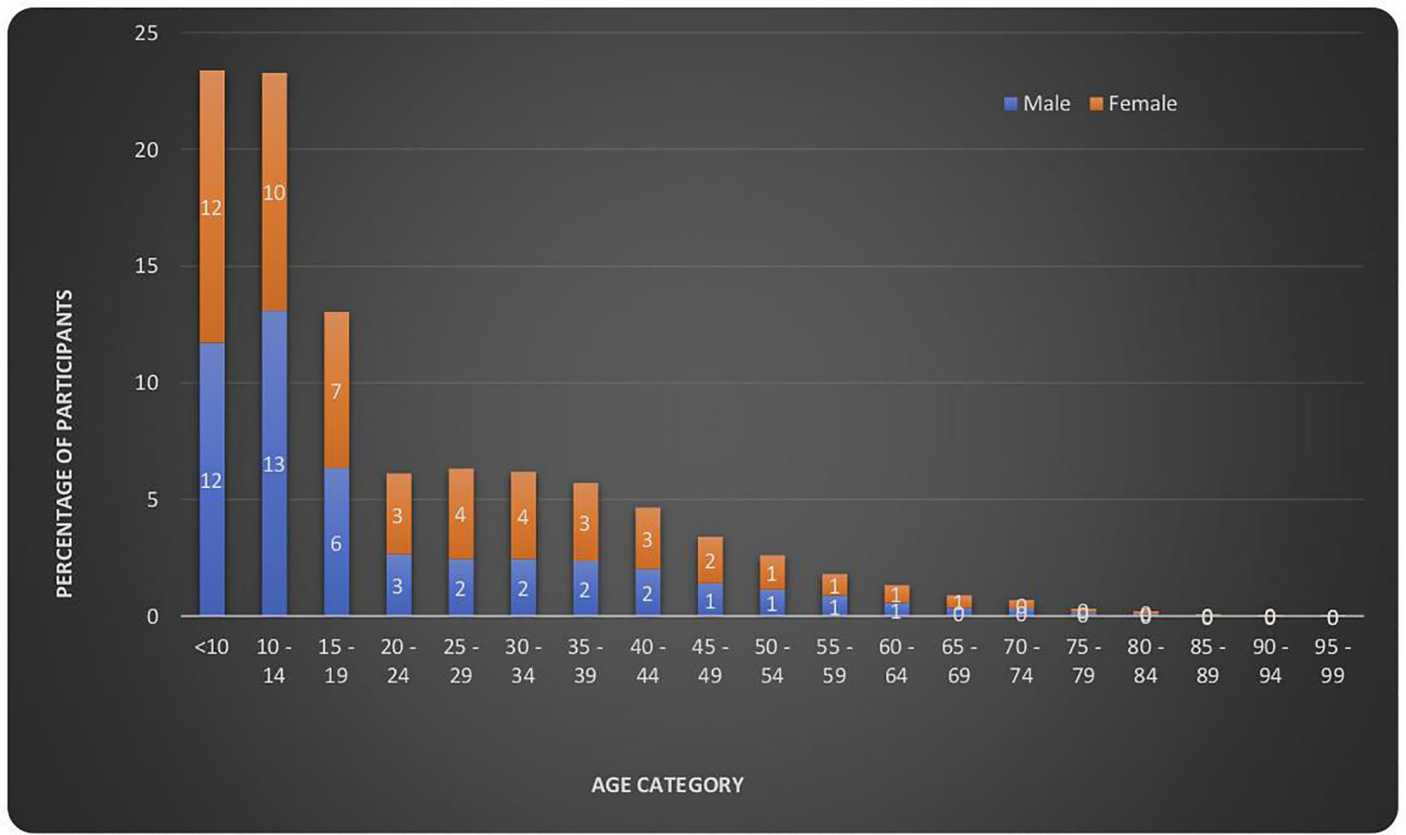
Distribution of participants by age and gender.

The age range of participants was 6–99 years, mean age ±Standard deviation (SD) was 22 ±16.2, median 15 (10–31). About 46% of the participants were less than 14 years old, 52% were females, 0.9% were currently smoking tobacco. Monthly household income was less than $50 in 46.7% of households (Table 1 and Fig 2).

**Table 1:** Socio-demographic characteristics of study participants across cities.

Characteristic	% Prevalence					
	All n = 20063	Lagos n = 11173	Kano n = 3808	Benin city n = 1931	Ilorin n = 1691	Enugu n = 1460
**Age group in years**						
**6–17**	56.8	58.6	68.0	34.9	36.5	35.8
**18–45**	33.1	33.1	22.0	45.8	48.7	49.8
**>45**	10.1	8.3	10.0	19.3	14.8	14.4
**Gender**						
**Female**	52.0	54.1	45.3	52.8	52.8	57.7
**Male**	48.0	45.9	54.7	47.2	47.2	42.3
**Highest level of education**						
**None**	6.8	1.2	18.9	8.4	11.7	5.8
**Primary**	29.4	32.7	26.7	22.1	22.5	21.2
**Faith based school**	8.0	1.3	30.5	0.2	1.5	0.1
**Some secondary**	21.9	28.5	10.6	11.2	16.5	18.7
**Completed secondary**	23.4	30.0	7.1	25.0	20.4	23.3
**Technical post-secondary**	3.1	1.5	2.9	13.9	6.9	3.3
**Some university**	3.1	2.4	2.1	4.3	9.3	8.5
**University graduate**	3.9	2.2	1.1	13.8	10.9	16.0
**Postgraduate**	0.4	0.2	0.2	1.1	0.3	2.5
**Employment status**						
**Self employed**	31.8	37.5	6.6	54.6	47.8	33.2
**Unemployed**	2.2	1.2	3.6	2.4	3.0	6.5
**Retired/ not working**	1.0	0.6	1.1	2.6	1.3	2.0
**Student**	60.1	59.5	72.7	38.9	46.5	53.3
**Stay at home parent**	4.8	1.1	15.9	1.4	1.4	4.0
**Disabled/ too ill to work**	0.1	0.1	0.1	0.1	0.0	1,1
**Monthly household income**						
**<$50**	46.6	30.2	93.1	42.5	34.8	40.0
**$50-$99**	11.8	12.6	3.6	17.3	25.2	20.0
**$100-$149**	24.2	35.6	2.3	17.5	14.1	12.2
**$150$199**	9.7	12.5	0.5	15.1	12.0	8.8
**$200-$245**	4.9	6.6	0.2	5.4	4.9	7.0
**≥$250**	2.8	2.5	0.4	2.0	8.9	12.0
Source of main cooking fuel*						
**Kerosene**	47.6	66.1	5.1	45.2	34.0	47.6
**LPG**	26.0	32.1	4.4	49.7	10.1	38.3
**Wood**	18.5	0.1	73.1	4.5	10.1	6.2
**Charcoal**	2.9	0.6	2.2	0.2	31.5	6.1
**Coal**	3.1	0.2	10.5	0.2	10.4	0.1
**Electricity**	0.7	0.7	0.0	0.2	3.9	1.4
**Agricultural waste**	0.0	0.0	3.2	0.0	0.0	0.0
**Current tobacco smokers**	0.9	0.8	0.5	1.0	1.4	1.0
#**Body mass index (kg/m**^**2**^**)**						
**Underweight**	6.2	8.1	0.8	1.4	5.8	5.7
**Normal**	53.7	58.0	43.5	39.3	55.6	51.3
**Overweight**	26.4	22.8	35.3	37.0	26.2	28.1
**Obese**	13.7	11.1	20.4	22.4	12.5	14.9

*0.4% gave no response

#Body mass index for 18 years and above.

### Prevalence of asthma and allergic rhinitis

**Fig 3:**
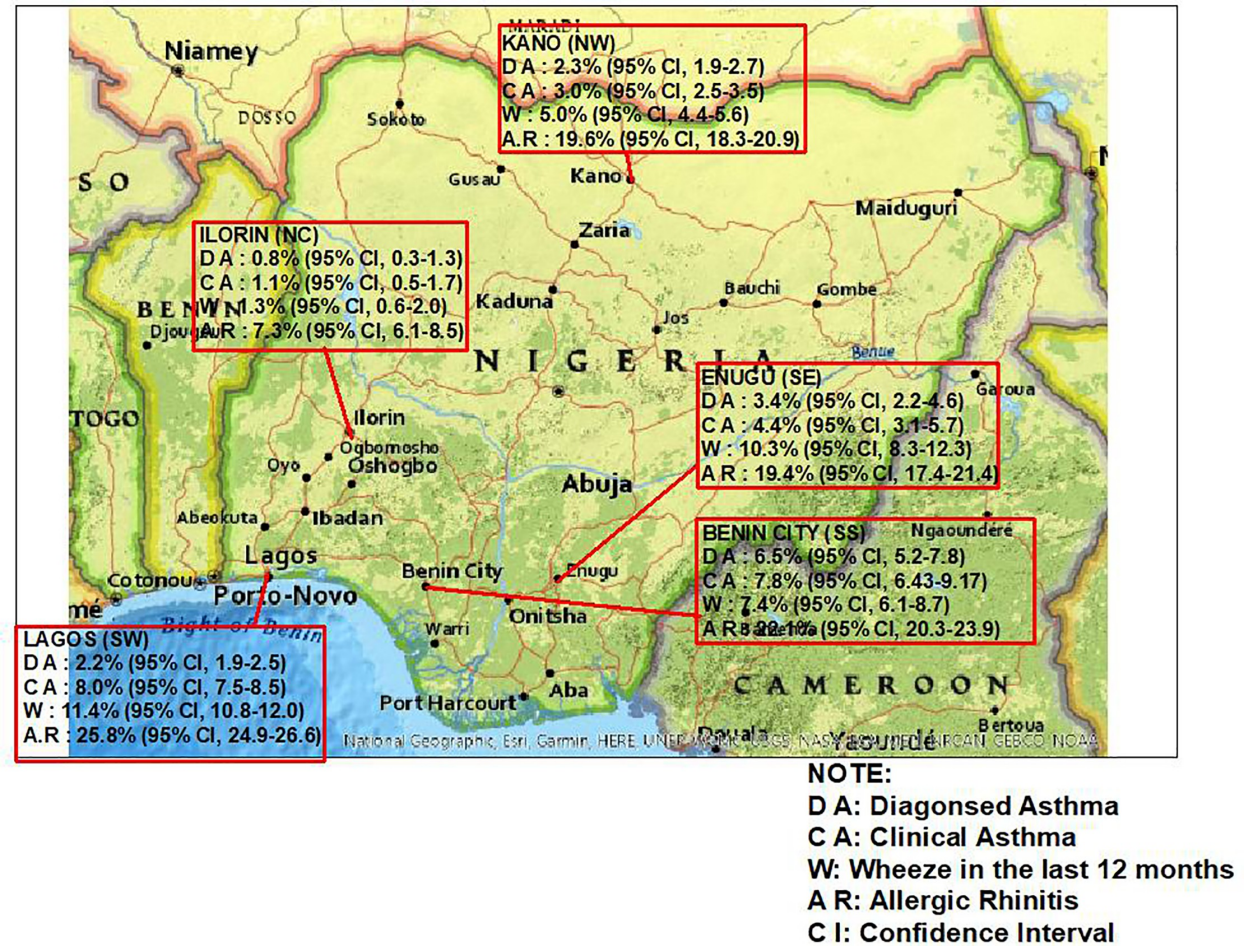
Map of Nigeria showing the prevalence of asthma and allergic rhinitis across cities. SW = South west, SE = South East, SS = South south, NW = North west. North central = North central. Map was created using USGS National Map Viewer (public domain): http://viewer.nationalmap.gov/viewer/.

The prevalence (95% confidence interval) of physician diagnosed asthma, clinical asthma and wheeze in the last 12 months among all participants was 2.5% (2.3–2.7), 6.4% (6.0–6.6) and 9.0% (8.6–9.4) respectively. The prevalence of allergic rhinitis was 22.8% (22.2–23.4). The prevalence of asthma and rhinitis varied across cities with the highest prevalence of clinical asthma and rhinitis of 8.0% and 25.8% respectively occurring in Lagos and the lowest of 1.1% and 7.3% respectively in Ilorin (Fig 3). The odd ratio (95% CI) for physician diagnosed asthma, clinical asthma, wheeze in the last 12 months and allergic rhinitis for residents of Lagos compared to residents of Ilorin was 2.52 (1.49–4.25), 7.30 (4.67–11.41), 9.35 (6.17–14.17) and 4.35 (3.61–5.24) respectively, 0.67 (0.47–0.87), 1.88 (1.45–2.43). 1.13 (0.94–1.35), and 1.44 (1.25–1.65) respectively compared to residents of Enugu, 0.94 (0.74–1.20), 2.83 (2.32–3.45), 2.44 (2.09–2.86) and 1.43 (1.30–1.56) respectively compared to residents of Kano and 0.32 (0.26–0.40), 1.04 (0.87–1.24), 1.61 (1.35–1.93) and 1.22 (1.09–1.37) respectively compared to residents of Benin-city.

The frequency of allergic rhinitis among persons with physician diagnosed asthma was 86.5% overall (89.3%, 86.5%, 85.6%, 74.2% and 88.9% for residents in Lagos, Kano, Benin city, Enugu and Ilorin respectively) and the frequency of physician diagnosed asthma among persons with allergic rhinitis was 10.2%. The frequency of allergic rhinitis among persons with clinical asthma was 74.7% and the frequency of clinical asthma among persons with allergic rhinitis was 20.9%. There was a significant association between the prevalence of allergic rhinitis and the prevalence of asthma (χ2 = 2120.6 p <0.0001)

### Prevalence of asthma and allergic rhinitis by age and gender distribution

Prevalence (95% CI) of physician diagnosed asthma, clinical asthma, wheeze in the last 12 months and allergic rhinitis was 4.1% (3.80–4.40), 10.0% (9.54–10.46), 14.0% (13.47–14.53), 26.3% (25.63–26.97) respectively among adults ≥18 years and 1.4% (1.25–1.55), 3.1% (2.88–3.32), 4.6% (4.34–4.87) and 18.9% (18.41–19.40) respectively among children 6–17 years. The Odds ratio (95% CI) for the diagnosis physician diagnosed asthma, clinical asthma, wheeze in the last 12 months and allergic rhinitis among adults compared to children was 2.98 (2.47–3.61), 3.54 (3.11–4.02). 3.35 (3.01–3.73) and 1.53 (1.43–1.64) respectively. Table 2 shows the prevalence of asthma by age and gender category.

**Table 2:** Percentage prevalence of asthma and allergic rhinitis across all age groups categorized by gender.

Asthma definitions	Age groups											
	6–17 years (95% CI)				18–45 years (95% CI)				>45 years (95% CI)			
	All n = 10948	Male n = 5690	Female n = 5258	OR* (95% CI)	All n = 6973	Male n = 2876	Female n = 4097	OR* (95% CI)	All n = 2142	Male n = 979	Female n = 1163	OR* (95% CI)
**Physician diagnosed asthma**	1.4 (1.2–1.6)	1.4 (1.1–1.7)	1.4 (1.1–1.7)	1.05 (0.77–1.44)	4.0 (3.5–4.5)	3.7 (3.0–4.4)	4.1 (3.5–4.7)	0.89 (0.70–1.14)	4.4 (3.5–5.3)	4.9 (3.5–6.3)	4.0 (2.9–5.1)	1.22 (0.81–1.85)
**Clinical asthma**	3.1 (2.9–3.3)	3.0 (2.6–3.4)	3.1 (2.6–3.6)	0.99 (0.80–1.24	9.8 (9.1–10.5)	10.0 (8.9–11.1)	9.7 (8.8–10.6)	1.03 (0.88–1.21)	10.7 (9.4–12.0)	11.8 (9.8–13.8)	9.7 (8.0–11.4)	1.25 (0.95–1.64)
**Wheeze in the last 12 months**	4.6 (4.3–4.9)	4.6 (4.1–5.1)	4.6 (4.0–5.2)	1.0 (0.83–1.19)	13.7 (12.9–14.5)	13.9 (12.6–15.2)	13.5 (12.5–14.5)	1.03 (0.90–1.19)	15.1 (13.6–16.6)	15.9 (13.6–18.2)	14.2 (12.2–16.2)	1.14 (0.9–1.44)
**Allergic rhinitis**	18.9 (18.4–19.4)	20.3 (19.3–21.3)	17.4 (16.4–18.4)	1.21 (1.10–1.33)	26.9 (25.9–27.9)	30.7 (29.0–32.4)	23.9 (22.6–25.2)	1.41 (1.27–1.57)	24.9 (23.1–26.7)	28.0 (25.2–30.8)	22.0 (19.6–24.4)	1.38 (1.13–1.68)

CI- Confidence Interval, OR = Odds ratio

*OR for males versus females

**Fig 4:**
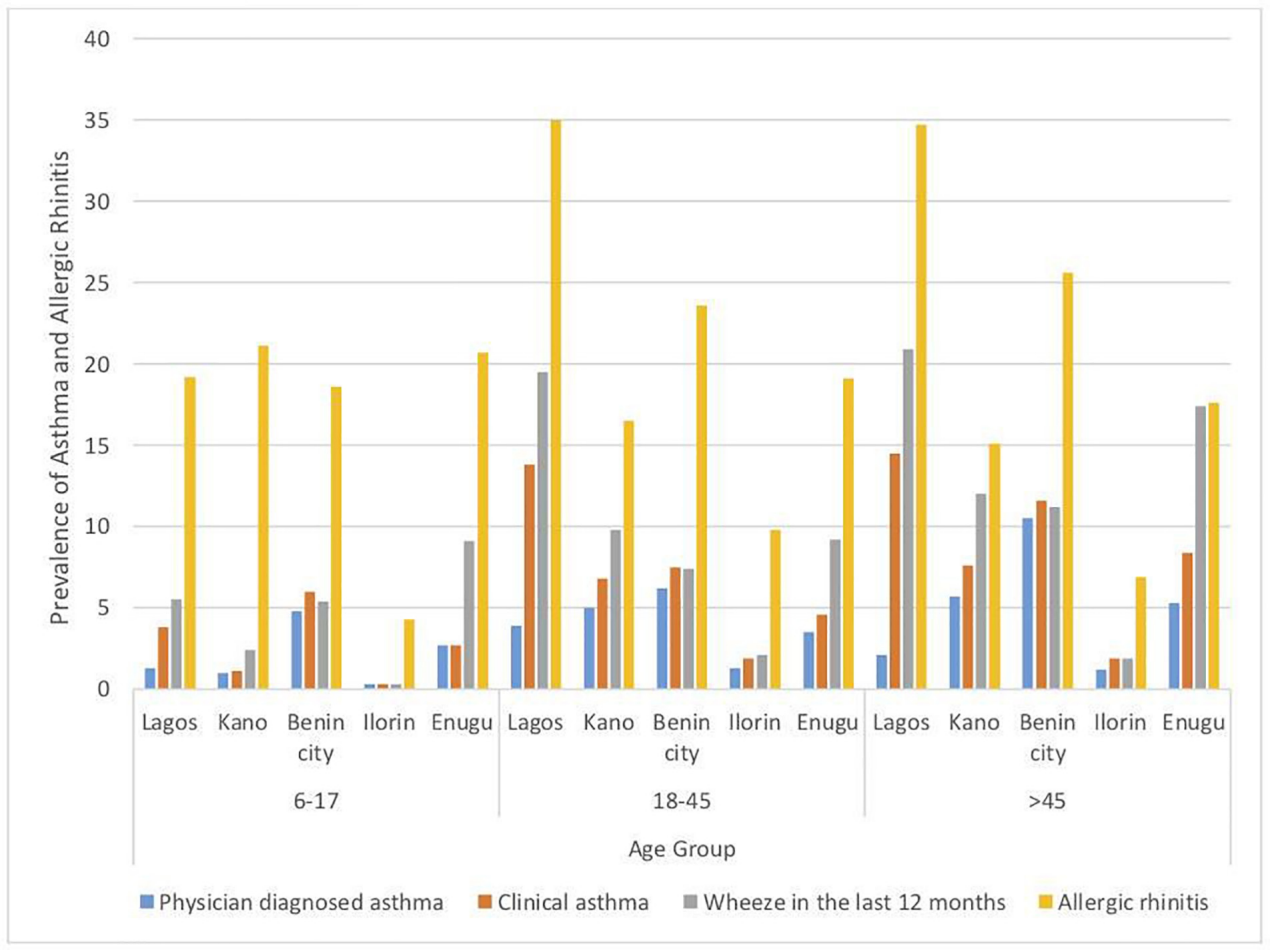
Percentage prevalence of asthma and allergic rhinitis across all age groups categorized by cities.

The prevalence of asthma and allergic rhinitis within age groups was also varied across cities with the highest prevalence of clinical asthma in children (6–17 years) and adults (18–45 years) occurring in Benin (6.0%) and Lagos (13.8%) respectively. (Fig 4).

The prevalence of physician diagnosed asthma, clinical asthma, wheeze in the last 12 months and allergic rhinitis was 1.2%, 2.2%, 2.7% and 17.3% respectively among 6-7-year olds, 2.1%, 3.7%, 5.5% and 20.4% respectively among 13-14-year olds and 1.6%, 4.1%, 6.7% and 19.7% respectively among adolescents 10-19-year olds. (Table 3)

**Table 3:** Percentage prevalence of asthma and allergic rhinitis across selected age groups among children and adolescents categorized by gender.

Asthma definition	Age group											
	6–7 years (95% CI)				13–14 years (95% CI)				10–19 years (95% CI)			
	All n = 2123	Male n = 1043	Female n = 1081	*OR (95% CI)	All n = 1437	Male n = 808	Female n = 629	*OR (95% CI)	All n = 7082	Male n = 3724	Female n = 3358	*OR (95% CI)
**Physician Diagnosed asthma**	1.2 (0.7–1.7)	1.4 (0.7–2.1)	1.1 (0.5–1.7)	1.21 (0.56–2.64)	2.1 (1.4–2.8)	2.0 (1.0–3.0)	2.4 (1.2–3.6)	0.83 (0.41–1.69)	1.6 1.3–1.9)	1.3 (0.9–1.7)	1.9 (1.4–2.4)	0.72 (0.50–1.04)
**Clinical asthma**	2.2 (1.6–2.8)	2.5 (1.6–3.5)	1.8 (1.0–2.6)	1.43 (0.79–2.60)	3.7 (2.7–4.7)	4.0 (2.6–5.4)	3.9 (2.4–5.4)	0.93 (0.54–1.60)	4.1 (3.6–4.6)	3.5 (2.9–4.1)	4.7 (4.0–5.4)	0.77 (0.61–0.98)
**Wheeze in the last 12 months**	2.7 (2.0–3.4)	2.9 (1.9–3.9)	2.6 (1.6–3.6)	1.12 (0.66–1.88)	5.5 (4.3–6.7)	4.7 (3.2–6.2)	6.7 (4.7–8.7)	0.69 (0.44–1.08)	6.7 (6.1–7.3)	6.3 (5.5–7.1)	7.1 (6.2–8.0)	0.84 (0.69–1.01)
**Allergic rhinitis**	17.3 (15.7–18.9)	18.7 (16.3–21.1)	15.9 (13.7–18.1)	1.22 (0.97–1.52)	20.4 (18.3–22.5)	22.2 (19.3–25.1)	17.7 (14.7-20-7)	1.34 (1.03–1.74)	19.7 (18.8–20.6)	20.8 (19.2–22.1)	18.4 (17.1–19.7	1.20 (1.07–1.35)

CI- Confidence Interval, OR = Odds ratio

*OR for asthma males versus females

**Fig 5:**
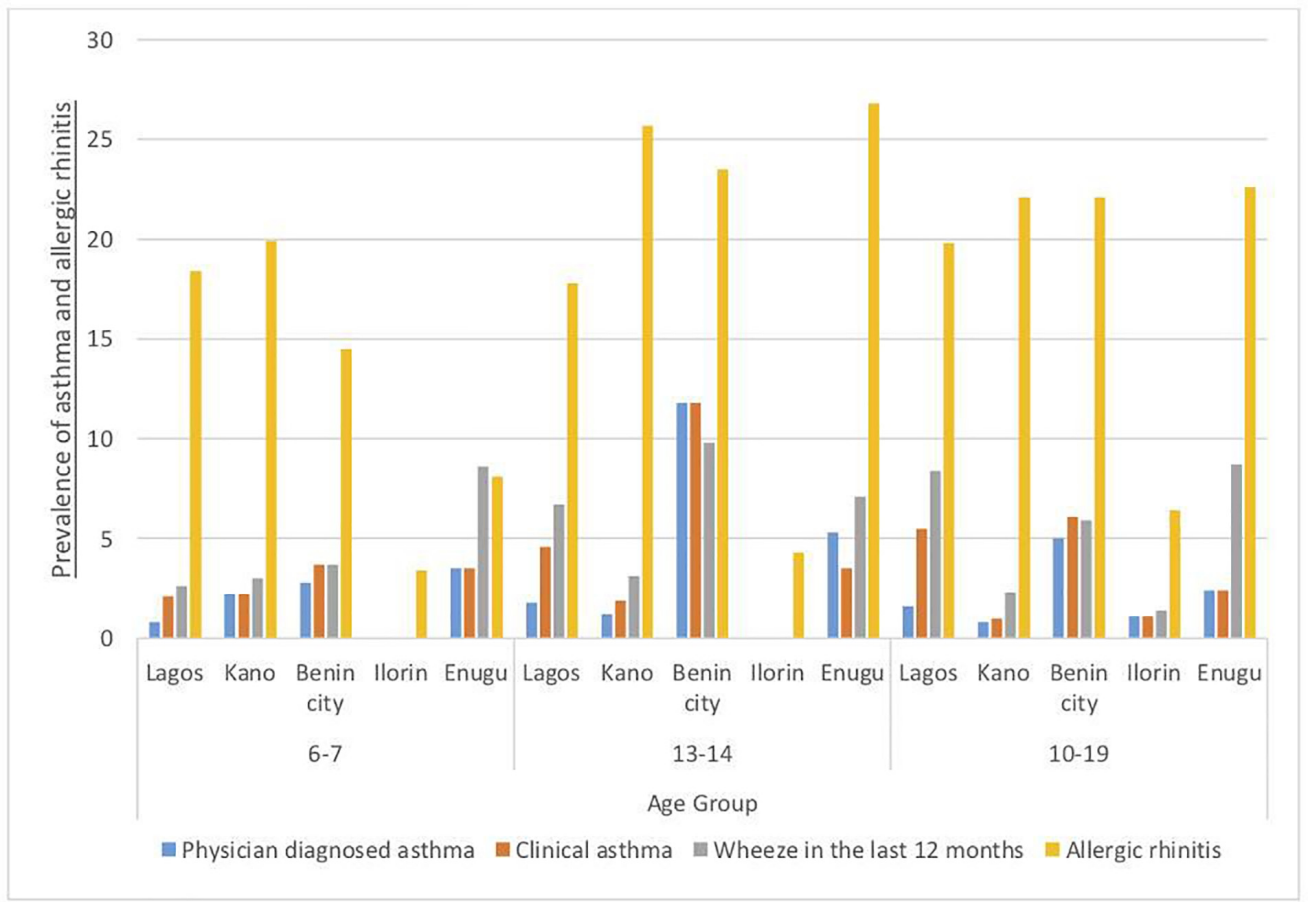
Percentage prevalence of asthma and allergic rhinitis across selected age groups among children and adolescents across cities categorized by cities.

The prevalence within these specific age groups also varied across cities with the highest prevalence of wheeze in the last 12 months among those 6-7-year old and those 13-14-year old found in Enugu (8.6%) and Benin (9.8%) respectively. (Fig 5)

### Characteristics of adult participants with and without clinical asthma

Among adult participants (≥ 18 years) with clinical asthma, 72.6% had allergic rhinitis, 3.3% were current smokers and 25% had a family history of asthma. More than half had a monthly household income less than $100 (Equivalent to about two times monthly minimum wage) and about three quarters used unclean cooking fuels. (Table 4).

**Table 4:** Comparison of socio-demographic characteristics among adults (≥18 years) with clinical asthma and those without.

Characteristics	With clinical asthma n = 913 (%)	Without clinical asthma n = 8202 (%)	p value
**Current tobacco smoking**	3.3	1.9	0.005
**Allergic rhinitis**	72.6	21.1	<0.001
**Family history of asthma**	25.1	6.1	<0.001
**Level of education**			
**None**	3.0	6.3	<0.001
**Primary**	10.2	9.1	
**Faith based school**	3.4	3.7	
**Some secondary**	8.4	7.9	
**Completed secondary**	52.5	45.4	
**Technical post-secondary**	6.7	7.8	
**Some university**	4.9	8.0	
**University graduate**	9.4	10.8	
**Postgraduate**	1.5	1.0	
**Body mass index (kg/m**^**2**^**)**			
**Underweight**	8.3	6.4	0.004
**Normal**	53.5	49.7	
**Overweight**	24.0	27.2	
**Obese**	14.2	16.7	
**Monthly household income**			
**<$50**	34.5	39.1	0.002
**$50-$99**	21.5	19.8	
**$100-$149**	26.3	21.0	
**$150$199**	10.1	10.9	
**$200-$245**	4.3	5.0	
**≥$250**	3.4	4.2	
Source of cooking fuel*			
**Kerosene**	56.6	49.8	<0.001
**LPG**	23.2	27.7	
**Wood**	6.8	12.0	
**Charcoal**	2.3	5.7	
**Coal**	1.4	3.2	
**Electricity**	0.4	1.0	
**Agricultural waste**	0.1	0.4	

*0.2% gave no response, p values represent the comparison between the reported proportions among persons with clinical asthma and those without clinical asthma.

Table 5 shows the multivariate logistic regression model for the determinants of current asthma among adults. We included all the variables that were significantly different among participants with current asthma and those without. Presence of allergic rhinitis, family history of asthma, current tobacco smoking and being underweight were independent determinants of clinical asthma and increased the risk by 7.72 (95% CI 6.56–9.09), 3.42 (95% CI 2.77–4.21), 1.61 (95% CI 1.02–2.55) and 1.33 (95% CI 1.00–1.77) times respectively.

**Table 5:** Multivariate logistic regression analysis for determinants of current asthma among adults.

Variable	Odds ratio	95% confidence interval	p value
Tobacco smoking status			
Never Smoker	Reference	-	-
Former Smoker	1.18	0.71–1.95	0.52
**Current Smoker**	**1.61**	**1.02–2.55**	**0.04**
**Allergic rhinitis (Yes)**	**7.72**	**6.56–9.09**	**<0.001**
**Family history of asthma (Yes)**	**3.42**	**2.77–4.21**	**<0.001**
Level of education			
Postgraduate	Reference	-	-
None	0.76	0.34–1.70	0.50
Primary	1.04	0.51–2.13	0.92
Faith based	1.65	0.72–3.76	0.24
Some secondary	0.77	0.37–1.60	0.48
Completed secondary	0.83	0.42–1.64	0.59
Technical post-secondary	0.64	0.31–1.33	0.23
Some university	0.53	0.25–1.10	0.09
University graduate	0.78	0.39–1.56	0.48
Body mass index			
Normal	Reference	-	-
Obese	0.85	0.68–1.06	0.15
Overweight	0.87	0.72–1.04	0.13
**Underweight**	**1.33**	**1.00–1.77**	**0.05**
Monthly household income			
≥$250	Reference	-	**-**
< $50	1.094	0.70–1.72	0.70
$50 - $99	1.329	0.84–2.10	0.23
$100 - $149	1.399	0.89–2.20	0.15
$150 - $199	1.027	0.64–1.66	0.91
$200 - $249	1.027	0.60–1.77	0.92
Source of cooking fuel			
Electricity	Reference	-	**-**
Agric Waste	0.39	0.04–3.95	0.43
Wood	1.01	0.33–3.05	0.99
Charcoal	1.28	0.40–4.09	0.67
Coal	1.04	0.30–3.53	0.96
Kerosene	2.52	0.87–7.32	0.09
Gas	1.50	0.52–4.38	0.46

Bold text represent independent determinants.

## Discussion

This is the first, large, broad-based population survey in Nigeria that provides an updated nationally representative prevalence of asthma and allergic rhinitis. This present study found a high prevalence of asthma and allergic rhinitis in Nigeria with heterogeneity across cities and across age groups. The national prevalence of physician diagnosed asthma, clinical asthma and wheeze in the last 12 months across all age groups was 2.5%, 6.4% and 9.0% respectively. The prevalence of clinical asthma increased with age from about 3% in children to about 10% in adults and varied from about 1% in Ilorin to 8% in Lagos. The prevalence of allergic rhinitis was 22.8% with similar variations as found in asthma across cities and age groups. Compared to children, adults were more likely to have a diagnosis of asthma and allergic rhinitis. Also, residents of the most urbanized and polluted city of Lagos were more likely to have clinical asthma compared to residents of other cities. The frequency of co-existent allergic rhinitis among persons with clinical asthma was 74.7%. Presence of allergic rhinitis, family history of asthma, current tobacco smoking and being underweight were independent determinants of current asthma among adults. With the population of Nigeria approaching 200 million [11], the number of persons with clinical asthma in Nigeria (approximately 13 million) is likely to rank among the highest in Africa. This warrants the recognition of asthma as an important public health disease and calls for prioritization and investment in risk reduction strategies and capacity building for appropriate management.

The diagnosis of asthma is typically based on characteristic clinical features and demonstration of variable airflow obstruction on lung function testing. The feasibility of conducting large-scale studies that incorporates lung function testing may be far reaching and the use of validated questionnaires has been an acceptable method for estimating asthma prevalence [2]. Reported physician diagnosis of asthma which may be the most reliable definition of asthma using a questionnaire may have limitations; it may underestimate asthma prevalence particularly where there is limited access to healthcare services as occurs in many parts of sub-Saharan Africa [29]. On the other hand, asthma diagnosis based on symptoms alone may be nonspecific and overestimate asthma prevalence, more likely in low and middle-income countries with widespread low levels of health literacy [30]. As a consequence of these limitations, we reported the prevalence of asthma based not only on physician diagnosis and characteristic symptoms (wheeze in the last 12 months) respectively, but included the prevalence of clinical asthma as also reported in the WHS [27]. Clinical asthma is likely to provide a more reliable estimate of asthma in Nigeria as well as other developing countries because it is reasonable to expect that persons with recurrent symptoms due to asthma are likely to find treatment regardless of physician diagnosis. In Nigeria and many parts of sub-Saharan Africa, prescription medications are usually obtainable across the counter without prescriptions, implying that persons with asthma symptoms may obtain asthma treatment without a diagnosis.

The low prevalence of physician diagnosed asthma among adults (4%) in this study is similar to the prevalence in many African countries, but lower than that in most Western European countries such as Australia and Sweden (20%) [27]. The lower prevalence of physician diagnosed asthma compared to clinical asthma in most African countries is likely to relate to limitations in access to health care services as we previously alluded to [13–14,31–32]. In Nigeria, low universal health coverage [33] particularly when put into the context of the very low earning ($100 monthly) by most households is an important constraint to accessing healthcare [34].

We note that the disparity between physician diagnosed asthma and clinical asthma is more profound in Lagos compared to other cities. Only about 30% of persons with clinical asthma in Lagos had a physician diagnosis compared to over 70% in other cities. This may signify differences between the pattern of health seeking behavior among inhabitants of Lagos compared to other cities. In Lagos, challenges such as high transportation and health service costs may lead to greater utilization of pharmacies and other medicine outlets for symptomatic treatment [35]. Health seeking behavior and utilization of healthcare services is associated with proximity to health facility [36,37]; in a previous study in Benin city, over 70% of households sought healthcare in hospitals [37].

The high prevalence of clinical asthma among adults (9.8%) in this present study aligns with existing data from previous meta-analysis [24], but higher than the average African prevalence (4.2%) [27]. Explanations for the higher prevalence of adult clinical asthma in Nigeria compared to most African countries may be related to higher cumulative exposure to air pollution [7,38–40]. The national mean annual concentration of ambient air particulate matter (PM_2.5_) of 122 μg/m in Nigeria ranks 6^th^ highest worldwide [38]. Similarly, the variability in asthma prevalence across Nigerian cities may also reflect the differences in the level of air pollution across these cities. For example, the higher prevalence of clinical asthma in Lagos and Benin-city where there is high traffic related air pollution compared to smaller cities such as Ilorin and Enugu support this [41]. The low prevalence of clinical asthma in smaller cities is consistent with a previous report from another small town of Ile-Ife, South-West Nigeria. [14]. Although not shown to be independently associated with clinical asthma in this study, air pollution from rampant use of unclean cooking fuels in Nigeria is likely to contribute to increased levels of household air pollution and ambient air pollution [42]. The significant association between current tobacco smoking and clinical asthma supports the role of respiratory exposures in increasing asthma risk. This emphasizes the need for the implementation of the tobacco control law and other policies that cutback emissions to improve air quality.

The overall prevalence of wheeze in this study among children 6–17 years of age (4.6%) is lower than the prevalence (15%) in black African children [12,15–16,43–44]. The prevalence among those 6-7-years old and those 13-14-years old of 2.7% and 5.5% respectively is lower than in the ISAAC study in Ibadan of 5.1% and 13% respectively [12]. However, the prevalence in specific cities such as Benin and Enugu are comparable to the Ibadan study. This highlights within country variations and brings to the fore the value of broad-based studies for estimating national prevalence. Reasons for the lower prevalence of asthma among Nigerian children may be related to variations in genetic modulators of asthma risk that exist within populations [45]. We are unaware of genome-wide studies regarding asthma risk in Africa which may enhance understanding of the disparities in asthma prevalence and also allow postulations on the potential influence of environment in modulating this risk. The trend we have reported on the increase in prevalence of asthma from childhood through adolescence to adulthood is consistent with the ISAAC study in Nigeria in which the prevalence of asthma was higher among older children [12, 43]. The increase in prevalence of asthma as the population ages highlights the need for further research on the putative role of the gene-environment interaction on the risk of developing asthma in this region.

This present study also demonstrates a high prevalence of allergic rhinitis (22.8%) comparable to previous reports in Nigeria and other parts of the world (9%-29.6%) [14,46–47]. We also substantiate a significant association between the co-existence (70–80%) of allergic rhinitis with asthma and the increased risk of asthma among persons with allergic rhinitis. [8–9,46–47]. Co-existent allergic rhinitis is known to worsens asthma control making it pertinent to recognize and treat both conditions [48].

The prevalence of asthma in this study was highest among the oldest participants >45 years and we recognize that persons with chronic obstructive pulmonary disease who may report similar symptoms and medication use as persons with asthma may have been included. This is in keeping with the higher prevalence of physician diagnosed asthma in the WHS when older adults (18–99 years) were included (6%) compared to when younger adults (18–45 years) were surveyed (4%) [27,49]. Despite the potential confounding effect of misdiagnosis, this finding is important because it highlights a high burden of chronic respiratory diseases among older adults. This informs healthcare delivery planning on the need to strengthen respiratory care services in Nigeria across the life course. This is even more relevant when put into the context of the projected population expansion in Nigeria by 2030 due to improved life expectancy with an increase in the proportion of the elderly population [11]. We recognize that the non-inclusion of participants from rural areas in this study as per the AIR survey protocol may limit the generalizability of our findings. However, recent surveys both nationally and globally report comparable asthma prevalence in urban and rural areas [27,50]. For example, in the study in Oyo state, South West Nigeria, the prevalence of asthma among 13-14-year-old children was 7.5% in the rural schools and 8.0% in the urban schools [50]. Although we translated the study questionnaires into local languages by standard methods and made adjustment during the pilot study, the lack of a formal validation process may be a limitation. This is because understanding asthma symptoms, particularly ‘wheeze’ may still be challenging despite translation which led to the incorporation of demonstration videos into the ISAAC studies. Finally, we recognize that fear of stigma and cultural beliefs may influence disclosure of health conditions in many African settings and may have had a marginal influence on the reported prevalence.

## Conclusions

In conclusion, we have demonstrated a high prevalence of asthma and allergic rhinitis in Nigeria with variations across cities and age groups. The high prevalence of asthma and allergic rhinitis compels a call to action on all stakeholders, policy makers and researchers to develop and implement population wide strategies to reduce environmental and lifestyle risks associated with these related conditions. There is also a need to strengthen the healthcare delivery system in Nigeria to facilitate access to efficient, effective and affordable diagnostic and treatment services.

## Supporting information

S1 File AIRDataoriginal. (SAV)

## References

[1] Vos T, Allen C, Arora M, Barber RM, Bhutta ZA, Brown A, et al Global, regional, and national incidence, prevalence, and years lived with disability for 310 diseases and injuries, 1990–2015: a systematic analysis for the Global Burden of Disease Study 2015. Lancet. 2016;388:1545–1602. 10.1016/S0140-6736(16)31678-6 27733282 PMC5055577

[2] Soriano JB, Abajobir AA, Abate KH, Abera SF, Agrawal A, Ahmed MB et al Global, regional, and national deaths, prevalence, disability-adjusted life years, and years lived with disability for chronic obstructive pulmonary disease and asthma, 1990–2015: a systematic analysis for the Global Burden of Disease Study 2015. The Lancet Respiratory Medicine. 2017;5:691–706. 10.1016/S2213-2600(17)30293-X 28822787 PMC5573769

[3] Asher MI, Montefort S, Björkstén B, Lai CKW, Strachan DP, Weiland SK, et al Worldwide time trends in the prevalence of symptoms of asthma, allergic rhinoconjunctivitis, and eczema in childhood: ISAAC Phases One and Three repeat multicounty cross-sectional surveys. Lancet. 2006;368:733–743. 10.1016/S0140-6736(06)69283-0 16935684

[4] Stewart AW, Mitchell EA, Pearce N, Strachan DP, Weiland SK on behalf of the ISAAC Steering Committee. The relationship of per capita gross national product to the prevalence of symptoms of asthma and other atopic diseases in children (ISAAC). Int J Epidemiol. 2001;30:173–179. 10.1093/ije/30.1.173 11171881

[5] Ellwood P, Asher MI, García-Marcos L, Williams H, Keil U, Robertson C, et al Do fast foods cause asthma, rhinoconjunctivitis and eczema? Global findings from the International Study of Asthma and Allergies in Childhood (ISAAC) Phase Three. Thorax. 2013;68:351–360. 10.1136/thoraxjnl-2012-202285 23319429

[6] Burney P, Jarvis D, Perez-Padilla R. The global burden of chronic respiratory disease in adults. Int J Tuberc Lung Dis. 2015;19:10–20. 10.5588/ijtld.14.0446 25519785

[7] Brauer M, Hoek G, Van Vliet P, Meliefste K, Fischer PH, Wijga A, et al Air pollution from traffic and the development of respiratory infections and asthmatic and allergic symptoms in children. Am J Respir Crit Care Med. 2002;166:1092–1098. 10.1164/rccm.200108-007OC 12379553

[8] Khan DA. Allergic rhinitis and asthma: epidemiology and common pathophysiology. Allergy Asthma Proc. 2014;35:357–361. 10.2500/aap.2014.35.3794 25295802

[9] Bousquet J, Khaltaev N, Cruz AA, Denburg J, Fokkens WJ, Togias A. Allergic Rhinitis and its Impact on Asthma (ARIA) 2008 update (in collaboration with the World Health Organization, GA(2)LEN and AllerGen). Allergy. 2008;63:8–160. 10.1111/j.1398-9995.2007.01620.x 18331513

[10] Adeloye D, Chan KY, Rudan I, Campbell H. An estimate of asthma prevalence in Africa: a systematic analysis. Croat Med J. 2013;54:519–531. 10.3325/cmj.2013.54.519 24382846 PMC3893990

[11] United Nations. World Population Prospects 2017 revision: Key findings and advance tables. Available from: https://www.compassion.com/multimedia/world-population-prospects.

[12] Falade AG, Olawuyi JF, Osinusi K, Onadeko BO. Prevalence and severity of symptoms of asthma, allergic rhinoconjunctivitis, and atopic eczema in 6- to 7-year-old Nigerian primary school children: the international study of asthma and allergies in childhood. Med Princ Pract. 2004;13:20–25. 10.1159/000074046 14657614

[13] Desalu OO, Oluboyo PO, Salami AK. The prevalence of bronchial asthma among adults in Ilorin, Nigeria. Afr J Med Med Sci. 2009;38:149–154. 20175418

[14] Obaseki DO, Awoniyi FO, Awopeju OF, Erhabor GE. Low prevalence of asthma in sub Saharan Africa: a cross sectional community survey in a suburban Nigerian town. Respir Med. 2014;108:1581–1588. 10.1016/j.rmed.2014.09.022 25443397

[15] Adetoun MB, Briggs DJ, Hansell AL. Prevalence of asthma and respiratory symptoms in children in a low socioeconomic status area of Nigeria. Int J Tuberc Lung Dis. 2013;17:982–988. 10.5588/ijtld.12.0434 23743319

[16] Ibeh CC, Ele PU. Prevalence of bronchial asthma in adolescent in Anambra State, Nigeria. Nigerian Journal of Internal Medicine. 2002;5:23–26.

[17] Desalu OO, Sanya EO, Adeoti AO, Aderibigbe SA, Kolo PM. Impact of operational definitions on the predictors and prevalence of asthma estimates: experience from a university students’ survey and implications for interpretation of disease burden. Ethiop J Sci. 2018;28:725. 10.4314/ejhs.v28i6.7 PMC6308751 30607089

[18] Rabe KF, Adachi M, Lai CKW, Soriano JB, Vermeire PA, Weiss KB, et al Worldwide severity and control of asthma in children and adults: The global asthma insights and reality surveys. J Allergy Clin Immunol. 2004;114:40–47. 10.1016/j.jaci.2004.04.042 15241342

[19] Khadadah M, Mahboub B, Al-Busaidi NH, Sliman N, Soriano JB, Bahous J. Asthma insights and reality in the Gulf and the near East. Int J Tuberc Lung Dis. 2009;13:1015–1022. 19723383

[20] Nafti S, Taright S, El Ftouh M, Yassine N, Benkheder A, Bouacha H, et al Prevalence of asthma in North Africa: The Asthma Insights and Reality in the Maghreb (AIRMAG) study. Respir Med. 2009;103:S2–S11. 10.1016/S0954-6111(09)70022-8 20122625

[21] Toren K, Brisman J, Jarvholm B. Asthma and asthma-like symptoms in adults assessed by questionnaires -a literature review. Chest. 1993;104:600–608. 10.1378/chest.104.2.600 7802735

[22] Burney P, Chinn S, Jarvis D, Luczynska C, Lai E. Variations in the prevalence of respiratory symptoms, self-reported asthma attacks, and use of asthma medication in the European Community Respiratory Health Survey (ECRHS). EurRespir J. 1996;9:687–695. 10.1183/09031936.96.09040687 8726932

[23] National Population Commission. Nigeria Population Census: State Population, 2006. Available from: http://nigeria.opendataforafrica.org/ifpbxbd/state-population-2006.

[24] Musa B., Aliyu M. Asthma prevalence in Nigerian adolescents and adults: systematic review and meta-analysis. African J Respir Med. 2014;10:4–9.

[25] Annesi-Maesano I, Didier A, Klossek M, Chanal I, Moreau D, Bousquet J. The score for allergic rhinitis (SFAR): A simple and valid assessment method in population studies. Eur J Allergy Clin Immunol. 2002;57:107–114. 10.1034/j.1398-9995.2002.1o3170.x 11929412

[26] Garrow JSWJ. Quetelet’s index (W/H2) as a measure of fatness. Int J Obes. 1985;9: 147–153. 4030199

[27] To T, Stanojevic S, Moores G, Gershon AS, Bateman ED, Cruz AA, et al Global asthma prevalence in adults: Findings from the cross-sectional world health survey. BMC Public Health. 2012;12:204–211. 10.1186/1471-2458-12-204 22429515 PMC3353191

[28] World Health Organization. Maternal, newborn, child and adolescent health; Health for the world’s adolescents: A second chance in the second decade. Available at www.who.int/adolescent/second-decade.

[29] Onyedum C, Ukwaja K, Desalu O, Ezeudo C. Challenges in the management of bronchial asthma among adults in Nigeria: a systematic review. Ann Med Health Sci Res. 2013;3:324–329. 10.4103/2141-9248.117927 24116307 PMC3793433

[30] Adekoya-Cole TO, Akinmokun OI, Enweluzo GO, Badmus OO, Alabi EO. Poor Health Literacy in Nigeria: Causes, Consequences and Measures to improve it. Nig Q J Hosp Med. 2015;25:112–117. 27295830

[31] Obel KB, Ntumba KJM, Kalambayi KP, Zalagile AP, Kinkodi KD, Munogolo KZ. Prevalence and determinants of asthma in adults in Kinshasa. PLoS One. 2017;12:1–13. 10.1371/journal.pone.0176875 PMC5413054 28464036

[32] Morgan BW, Siddharthan T, Grigsby MR, Pollard SL, Kalyesubula R, Wise RA, et al Asthma and allergic disorders in Uganda: A population-based study across urban and rural settings. J Allergy Clin Immunol Pract. 2018;6:1580–1587. 10.1016/j.jaip.2017.11.032 29361510 PMC6050146

[33] Uzochukwu BC, Ughasoro MD, Etiaba E, Okwuosa C, Envuladu E, Onwujekwe O. Health care financing in Nigeria: Implications for achieving universal health coverage. Niger J Clin Pract. 2015;18:437–444. 10.4103/1119-3077.154196 25966712

[34] Poverty & Equity Data Portal [Internet]. The World Bank. 2018. Available from: http://povertydata.worldbank.org/poverty/country/NGA.

[35] Dave-AgboolaI O, Raji JI. Health-seeking behavior of malaria patients in Lagos, Nigeria. Int J Health Sci Res. 2018;8:259–264

[36] Omotoso O. Health seeking behavior among the rural dwellers in Ekiti State, Nigeria. African Research Review. 2010; 4:125–138

[37] Adams VY, Aigbokhaode AQ. Healthcare seeking behavior of heads of households in an urban community in South South Nigeria. Ann Biomed Sci 2015;14:121–130.

[38] PM2.5 air pollution, mean annual exposure (micrograms per cubic meter) [Internet]. The World Bank. 2016. Available from: https://data.worldbank.org/indicator/EN.ATM.PM25.MC.M3.

[39] World Health Organization. Exposure to ambient air pollution from particulate matter for 2016. Available at: http://www.who.int/airpollution/data/AAP_exposure_Apr2018_final.pdf?ua=1.

[40] Guarnieri M, Balmes JR. Outdoor air pollution and asthma. Lancet. 2014;383:1581–1592. 10.1016/S0140-6736(14)60617-6 24792855 PMC4465283

[41] Komolafe AA, Adegboyega SA, Anifowose AY, Akinluyi FO, Awoniran DR. Air pollution and climate change in Lagos, Nigeria: needs for proactive approaches to risk management and adaptation. Am J Environ Sci. 2014;10:412–423.

[42] Ozoh OB, Okwor TJ, Adetona O, Akinkugbe AO, Amadi CE, Esezobor C, et al Cooking fuels in Lagos, Nigeria: Factors associated with household choice of kerosene or Liquefied Petroleum Gas (LPG). Int J Environ Res Public Health. 2018;15:641. 10.3390/ijerph15040641 PMC5923683 29614713

[43] Falade AG, Ige OM, Yusuf BO, Onadeko MO, Onadeko BO. Trends in the prevalence and severity of symptoms of asthma, allergic rhinoconjunctivitis, and atopic eczema. J Natl Med Assoc. 2009;101:414–418. 10.1016/s0027-9684(15)30925-1 19476194

[44] Ait-Khaled N, Odhiambo J, Pearce N, Adjoh KS, Maesano IA, Benhabyles B, et al Prevalence of symptoms of asthma, rhinitis and eczema in 13- to 14-year-old children in Africa: The International Study of Asthma and Allergies in Childhood Phase III. Allergy. 2007;62:247–258. 10.1111/j.1398-9995.2007.01325.x 17298341

[45] Oliveira P, Costa GNO, Damasceno AKA, Hartwig FP, Barbosa GCG, Figueiredo CA, et al Genome-wide burden and association analyses implicate copy number variations in asthma risk among children and young adults from Latin America. Sci Rep. 2018;8:14475 10.1038/s41598-018-32837-w 30262839 PMC6160443

[46] Desalu OO, Salami AK, Isek KR, Oluboyo PO. Prevalence of self-reported allergic rhinitis and its relationship with asthma among adult Nigerians. J Investig Allergol Clin Immunol. 2009;19:474–480. 20128422

[47] Zhang Y, Zhang L. Increasing Prevalence of Allergic Rhinitis in China. Allergy Asthma Immunol Res. 2019;11:156–169. 10.4168/aair.2019.11.2.156 30661309 PMC6340797

[48] Reddel KH, Pedersen S. Global Initiative for Asthma. Global strategy for asthma management and prevention. 2018 Available from: www.ginasthma.org.

[49] Sembajwe G, Cifuentes M, Tak SW, Kriebel D, Gore R, Punnett L. National income, self-reported wheezing and asthma diagnosis from the World Health Survey. Eur Respir J. 2010;35:279–286. 10.1183/09031936.00027509 19741032

[50] Oluwole O, Arinola OG, Falade GA, Ige MO, Falusi GA, Aderemi T, et al Allergy sensitization and asthma among 13-14-year-old school children in Nigeria. Afr Health Sci. 2013;13:144–153. 10.4314/ahs.v13i1.20 23658581 PMC3645088

